# The chromosomal passenger complex (CPC) as a key orchestrator of orderly mitotic exit and cytokinesis

**DOI:** 10.3389/fcell.2015.00014

**Published:** 2015-03-05

**Authors:** Mayumi Kitagawa, Sang Hyun Lee

**Affiliations:** Program in Cancer and Stem Cell Biology, Duke-NUS Graduate Medical School SingaporeSingapore

**Keywords:** Aurora B kinase, chromosomal passenger complex, abscission, mitotic exit, cytokinesis, nuclear envelope reformation, chromosome condensation, chromosome segregation

## Abstract

Understanding the molecular network of orderly mitotic exit to re-establish a functional interphase nucleus is critical because disordered mitotic exit inevitably leads to genomic instability. In contrast to the mechanisms of the entrance to mitosis, however, little is known about what controls the orderly exit from mitosis, particularly in mammalian cells. The chromosomal passenger complex (CPC), which is composed of Aurora B, INCENP, Borealin and Survivin, is one of the most widely studied and highly conserved hetero-tetrameric complexes. The CPC orchestrates proper chromosome segregation with cytokinesis by targeting to specific locations at different stages of mitosis. Recent studies reveal that controlling CPC localization and Aurora B kinase activity also serves as a key surveillance mechanism for the orderly mitotic exit. This ensures the reformation of a functional interphase nucleus from condensed mitotic chromosomes by delaying mitotic exit and cytokinetic processes in response to defects in chromosome segregation. In this review, we will summarize the latest insight into the molecular mechanisms that regulate CPC localization during mitotic exit and discuss how targeting Aurora B activity to different locations at different times impacts executing multiple mitotic exit events in order and recently proposed surveillance mechanisms. Finally, we briefly discuss the potential implication of deregulated Aurora B in inducing genomic damage and tumorigenesis with current efforts in targeting Aurora B activity for anti-cancer therapy.

## Introduction

For accurate cell division, an exact copy of the genome must be equally transmitted from a mother cell to two dividing daughter cells. This requires equal segregation of the duplicated sister chromatids during mitosis followed by cytoplasmic division involving cytoskeletal reorganization and membrane scission events. These processes are tightly orchestrated by the opposing activities of protein kinases and phosphatases on mitotic chromosomes and in the cell equator, which includes the spindle midzone and the equatorial cortex. Such opposing activities are also likely present in the midbody to complete cytokinesis.

The dynamic localization of chromosomal passenger proteins in the proper time and space predicts the molecular connections of chromosome segregation and cytokinesis. These two events can be orchestrated by a set of master regulators, which are localized to a mitotic chromosome prior to its segregation but thereafter transferred to the cell equator for cytokinesis (Earnshaw and Bernat, 1991). This hypothesis was postulated from the identification of the inner centromere protein (INCENP) as the first passenger protein that resides in the inner centromere in early mitosis while it detaches from anaphase chromosomes and localizes in the spindle midzone and subsequently the equatorial cortex (Cooke et al., 1987). Later, it was shown that INCENP forms a complex with Aurora B kinase (Adams et al., 2000; Kaitna et al., 2000), which was known to be required for proper cell division. It is now recognized that the chromosomal passenger complex (CPC) is composed of the enzymatic core Aurora B kinase, the scaffold protein INCENP, and two other non-enzymatic subunits Survivin/BIRC5 and Borealin/CDCA8 (reviewed by Carmena et al., 2012). Aurora B interacts with the C-terminal region of INCENP called the IN-box domain. The N-terminal residues 1–58 containing the CEN-box of INCENP form a triple-helix bundle with Borealin and Survivin that is required for CPC localization to the inner centromere, the spindle midzone and the midbody (Ainsztein et al., 1998; Klein et al., 2006; Vader et al., 2006; Jeyaprakash et al., 2007). Aurora B kinase activity itself is also required for forcing CPC to localize to the inner centromere and the cell equator (Xu et al., 2009; Qian et al., 2013). Notably, as the stability of individual components of the CPC is supported by the protein-protein interactions within the CPC, genetic knockout or depletion of any of the CPC components causes similar phenotypes as the loss of Aurora B kinase activity (Adams et al., 2001; Honda et al., 2003; Gassmann et al., 2004; Klein et al., 2006; Vader et al., 2006).

The changes in CPC localization at different stages of mitosis and cytokinesis provide an effective means to restrict the phosphorylation of its substrates to the appropriate time and space during mitotic progression (reviewed by van der Horst and Lens, 2014). Starting from entry into mitosis, the CPC accumulates at the inner centromeres, which is a prerequisite for establishing a functional microtubule attachment to mitotic chromosomes by destabilizing erroneous kinetochore-microtubule attachment, activating the mitotic spindle assembly checkpoint (SAC) until accurate bipolar spindle attachment is achieved (also called amphitelic attachment) and promoting chromosome congression to the metaphase plate. The details on how the CPC together with other mitotic regulators controls chromosome alignment and SAC signaling during mitotic entry and metaphase completion have recently been reviewed (Funabiki and Wynne, 2013).

Upon the metaphase-to-anaphase transition, the CPC relocates from anaphase chromosomes to the cell equator where it promotes the initiation and ingression of the cleavage furrow, formation and stabilization of the spindle midzone and axial shortening of the segregating chromosome arms near the ingressing cleavage furrow. The CPC also controls the timing of nuclear envelope reformation (NER), and finally in the midbody, the CPC controls the timing of abscission that completes cytokinesis (Norden et al., 2006; Miyauchi et al., 2007; Mora-Bermudez et al., 2007; Ramadan et al., 2007; Hu et al., 2008; Maerki et al., 2009; Douglas et al., 2010; Neurohr et al., 2011; Capalbo et al., 2012; Carlton et al., 2012; Kitagawa et al., 2013; Afonso et al., 2014; Thoresen et al., 2014). How the CPC regulates and is regulated to execute these multiple mitotic events especially from the entrance into mitosis to anaphase onset has been extensively studied and briefly summarized here (for recent reviews, see also Carmena et al., 2012; van der Horst and Lens, 2014). This review mainly focuses on the molecular mechanisms in regulating CPC during mitotic exit and cytokinesis. We also focus on recent findings that reveal the CPC's role of surveillance in proper NER and chromosome decondensation during mitotic exit and completion of cytokinesis, thereby designating the CPC as a key guardian of genomic stability. Finally, we briefly discuss the implication of deregulated Aurora B in fuelling genomic instability and tumorigenesis with current efforts in targeting Aurora B for anti-cancer therapy.

## Review

### Multiple steps in CPC translocation from anaphase chromosomes to the cell equator

During the metaphase-to-anaphase transition, a population of the CPC leaves the inner centromeres and anaphase chromosome arms and transfers to the spindle midzone (Cooke et al., 1987; Carmena et al., 2012). Subsequently, the CPC is also transferred to the equatorial cortex (Earnshaw and Cooke, 1991; Murata-Hori and Wang, 2002), the region of the plasma membrane where the cleavage furrow is assembled (Fededa and Gerlich, 2012). Relocation of the CPC from anaphase chromosomes to the cell equator is a key landmark event for cytokinesis, which is coupled to the initiation of mitotic exit. In general, this event is facilitated by at least three measures: (1) the end of targeting the CPC to the chromosome arm and the centromere, (2) the removal of the CPC from the chromosome arm and the centromere, and (3) relocating and accumulating the CPC in the cell equator and midbody. This change in localization is key for orchestrating the orderly mitotic exit by suppressing Aurora B activity at the location (anaphase chromosome) where it is no longer needed and/or needs to be terminated while it promotes the gain of Aurora B activity at the new location (cell equator and midbody) where its function now becomes essential. For instance, targeting Aurora B to mitotic chromosomes from the entrance to mitosis promotes chromosome condensation (Ono et al., 2004; Lipp et al., 2007; Nakazawa et al., 2011; Tada et al., 2011). Conversely, active removal of the CPC from segregating anaphase chromosomes is required to re-establish the nucleus to a functional interphase state by promoting chromosome decondensation and NER.

#### The Mechanisms of Facilitating and Ending CPC Targeting to Anaphase Chromosomes and the Centromere

**Figure 1:**
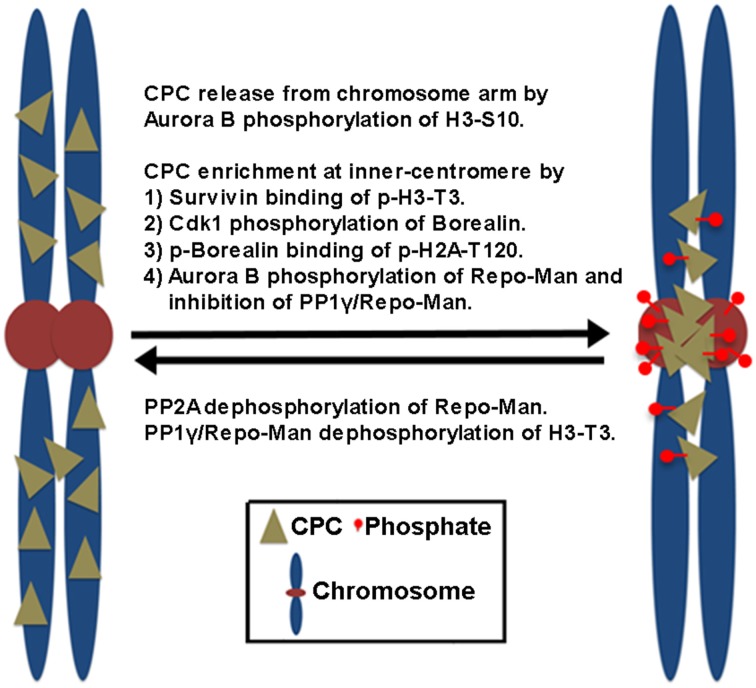
**The proposed mechanisms of CPC enrichment at the inner centromeres**. Aurora B-dependent phosphorylation of histone H3 on Ser10 (H3-S10) dissociates the CPC from the chromosome arm. Subsequently, the CPC enriches at the inner centromeres. This involves the direct and indirect interaction of Survivin and Borealin with the centromere-specific phosphorylated histone markers (p-H3-T3, p-H2A-T120) at the inner centromeres created by Haspin and Bub1 kinases. Cdk1 phosphorylation of Borealin is required for Borealin binding to the histone marker H2A-T120 phosphorylated by Bub1 kinase. In contrast, PP1γ/Repo-Man phosphatase acts antagonistically by dephosphorylating the histone makers at the chromosome arm. At the centromeres, Aurora B phosphorylation of Repo-Man on Ser893 prevents PP1γ/Repo-Man recruitment to histones, thereby the CPC is enriched at the inner centromeres. Conversely, PP2A reverses this inhibitory phosphorylation of Repo-Man by Aurora B.

In mammalian cells, the CPC is first found on pericentromeric heterochromatin during late S phase, and CPC targeting to heterochromatin involves heterochromatin protein 1 (HP1) binding to INCENP (Cooke et al., 1987; Ainsztein et al., 1998; Nozawa et al., 2010) while Aurora B-dependent phosphorylation of histone H3 on Ser10 (H3-S10) dissociates HP1 from trimethylated Lys9 of histone H3 (H3K9me3), which dissociates the CPC from the chromosome arm (Fischle et al., 2005; Hirota et al., 2005). Subsequently, the CPC enriches at the inner centromeres before the metaphase-to-anaphase transition, which depends on the direct and indirect interaction of Survivin and Borealin with the centromere-specific histone markers created by other mitotic kinases (Figure 1). Survivin binds the phosphorylated histone H3 on Thr3 (H3-T3) through Haspin kinase (Kelly et al., 2010; Wang, 2010). Borealin that has been phosphorylated by Cdk1 binds to the phosphorylated histone H2A on Thr120 (H2A-T120) through Bub1 kinase via the shugoshin protein (Kawashima et al., 2010; Tsukahara et al., 2010). These two histone phosphorylation markers seem to overlap at the inner centromeres, potentially explaining how the CPC becomes concentrated at this site (Yamagishi et al., 2010). In contrast, PP1γ/Repo-Man phosphatase acts antagonistically to Haspin and dephosphorylates H3-T3 at the chromosome arm but not at the centromere because Aurora B phosphorylation of Repo-Man on Ser893 at the centromere prevents PP1γ/Repo-Man recruitment to histones. Therefore, Aurora B activity also defines its own centromere targeting (Qian et al., 2013).

Upon the metaphase-to-anaphase transition, inhibiting chromosome targeting and releasing the CPC from the centromere require the protein phosphatases PP1 and PP2A in mammalian cells. PP2A reverses the inhibitory phosphorylation of Repo-Man on Ser893 by Aurora B (Qian et al., 2013). Therefore, reversing histone phosphorylation of H3-T3 and H2A-T120 upon anaphase onset, which is contributed by the activity of PP1γ/Repo-Man, leads to the suppression of CPC recruitment to chromosomes (Kelly et al., 2010; Qian et al., 2011; Vagnarelli et al., 2011), also releasing the CPC from the inner centromeres. Of note, Cdk1 is also responsible for the phosphorylation of Borealin and targeting the CPC to the inner centromeres (Kawashima et al., 2010; Tsukahara et al., 2010) while releasing of the CPC from the inner centromeres is initiated by decreasing Cdk1 activity. Therefore, it is equally possible that reversing Cdk1 phosphorylation of Borealin may also contribute to preventing the CPC from targeting to the inner centromeres through phosphorylated H2A-T120. However, CPC localization to the inner centromere does not seem to be a prerequisite step for CPC relocation to the cell equator because knockout of the condensin subunit SMC2 in DT40 cells inhibits CPC accumulation to the centromeres and maintains the CPC in the chromosome arms, but the CPC is still able to relocate to the cell equator upon anaphase onset (Hudson et al., 2003). Therefore, the chromosome arm is likely the major location where the CPC is removed from anaphase chromosomes and relocated to the cell equator after its release from the inner centromeres.

#### The Mechanisms of Removing the CPC from Anaphase Chromosomes

Ubiquitination of Aurora B contributes to the active removal of the CPC from the anaphase chromosome. Aurora B can directly interact with the E3 ubiquitin ligase complex Cul3-Kelch-like protein 21 (KLHL21) (Maerki et al., 2009). Aurora B is then ubiquitylated by two midzone-associated complexes, CUL3–KLHL9–KLHL13 (Sumara et al., 2007) and CUL3–KLHL21 (Maerki et al., 2009). Ubiquitylated Aurora B is subsequently removed from the anaphase chromosome by the AAA+ ATPase Cdc48 (cell division control protein 48; also known as p97) and its adaptor proteins Ufd1–Npl4 (Ramadan et al., 2007; Dobrynin et al., 2011). This process is also thought to contribute to the determination of the levels and distribution of the CPC on chromosomes before mitotic exit and support chromosome decondensation and NER after mitotic exit (Ramadan et al., 2007).

#### The Mechanisms of Relocating the CPC from Anaphase Chromosomes

**Figure 2:**
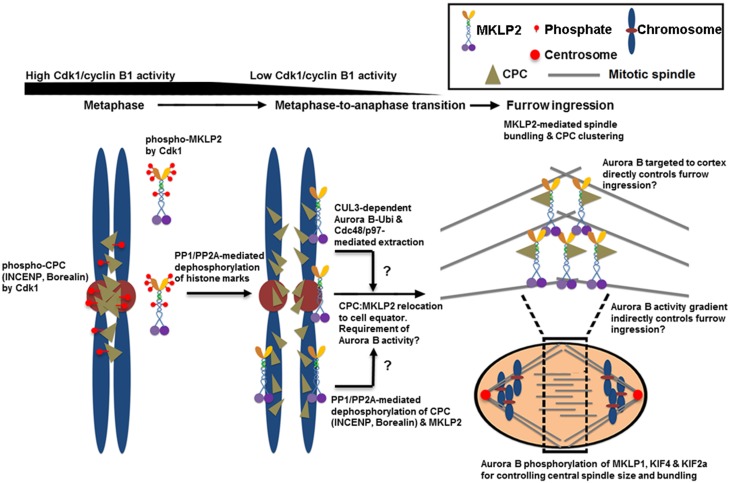
**The proposed mechanisms of CPC relocation from anaphase chromosomes to the cell equator, which promote the stability of the spindle midzone and furrow ingression**. Cdk1 phosphorylates multiple sites of INCENP, Borealin and MKLP2 in early mitosis. These phosphorylation events are required for targeting the CPC to the histone markers at the inner centromere that are phosphorylated by Haspin and Bub1 kinases (see Figure 1). Cdk1 phosphorylation is also required for inhibiting MKLP2's microtubule binding, oligomerization/clustering and recruitment to mitotic chromosomes. Upon anaphase onset, however, reversing Cdk1 phosphorylation and the histone markers by PP1 and PP2A phosphatases is necessary to release the CPC from and stop targeting the CPC to the inner centromere. The dephosphorylation of MKLP2 promotes its kinesin function to relocate the CPC from anaphase chromosomes to the cell equator, possibly via INCENP binding. The dephosphorylation of INCENP on Thr59 and sufficient Aurora B activity are also required for CPC relocation, but the underlying mechanisms remain unclear. Alternatively, the CPC is proposed to be removed from anaphase chromosomes via ubiquitination of the Aurora B by CUL3-KLHL9–KLHL13 and CUL3–KLHL21 E3 enzymes. Ubiquitylated Aurora B (and presumably the CPC) is removed from anaphase chromosomes by AAA+ ATPase Cdc48/p97 and its adaptor proteins Ufd1–Npl4. This process may contribute to the levels and distribution of the CPC on chromosomes even before anaphase onset, and it may support chromosome decondensation and NER in late anaphase. Whether MKLP2 and Cdc48/p97 collaborate to remove the CPC from anaphase chromosomes is unknown. In the cell equator, the involvement of MKLP2 in microtubule binding, bundling, and oligomerization/clustering may contribute to central spindle assembly/stabilization and clustering/activation of the CPC at the cell equator. This clustering event may also stably deliver the CPC close to the cell cortex for robust furrow ingression directly or indirectly via Aurora B phosphorylation gradients. In the spindle midzone, Aurora B also phosphorylates MKLP1, KIF4, and KIF2a to regulate central spindle size and bundle central spindles.

The CPC that is released from the inner centromere and the chromosome arm also needs to be actively relocated from anaphase chromosomes to the spindle midzone (Murata-Hori and Wang, 2002) and subsequently to the equatorial cortex (Earnshaw and Cooke, 1991). This relocation process requires the interaction of INCENP and Aurora B with the mitotic motor kinesin MKLP2 (Gruneberg et al., 2004; Cesario et al., 2006; Goto, 2006; Hummer and Mayer, 2009; Kitagawa et al., 2014) as well as Aurora B kinase activity (Xu et al., 2009) (Figure 2). The CPC and MKLP2 only interact during anaphase when Cdk1-mediated inhibitory phosphorylation is removed from INCENP on Thr59 and MKLP2 at multiple residues (Hummer and Mayer, 2009; Kitagawa et al., 2014). The increase in microtubule binding affinity of the CPC is also mediated in part by dephosphorylation of Thr59 of INCENP (Hummer and Mayer, 2009). MKLP2 is also essential for CPC relocation to the spindle midzone because RNAi-mediated knockdown of MKLP2 prevents CPC accumulation to the spindle midzone, leading to failed cytokinesis (Hill et al., 2000; Gruneberg et al., 2004). Similarly, the expression of an INCENP mutant in which Thr59 is mutated to a phosphomimetic glutamic acid prevents the CPC from localizing to the cell equator that leads to cytokinesis failure (Hummer and Mayer, 2009). In addition to INCENP, MKLP2 is also hyper-phosphorylated by Cdk1 prior to the metaphase-to-anaphase transition while it is rapidly dephosphorylated upon anaphase transition via PP1/PP2A (Kitagawa et al., 2014). Cdk1 phosphorylation of MKLP2 is essential for maintaining spindle dynamics that are required for chromosome congression during early mitotic progression because phosphoresistant, but not phosphomimetic, mutants of MKLP2 in early mitotic cells prematurely bind microtubules and alter spindle dynamics by enhancing microtubule stability (Kitagawa et al., 2014). Notably, Cdk1 phosphorylation of MKLP2 also controls the timely recruitment of MKLP2 to anaphase chromosomes (Kitagawa et al., 2014), but the chromosome adaptor that recruits MKLP2 is unknown. When the stalk domain of MKLP2 that is responsible for its chromosome targeting is ectopically expressed, it blocks CPC relocation from anaphase chromosomes, suggesting that MKLP2 may be responsible for removing the CPC directly from anaphase chromosomes (Kitagawa et al., 2014). How the spatiotemporal recognition between MKLP2 and the CPC occurs on anaphase chromosomes remains unclear. Furthermore, because MKLP2 has a strong microtubule bundling activity that is suppressed by Cdk1 phosphorylation, it is unclear how this chromosome targeting of MKLP2 upon anaphase onset occurs before its mitotic spindle binding and bundling.

The interaction between MKLP2 and the CPC is mediated by the C-terminal cargo-binding domain of MKLP2 (Hummer and Mayer, 2009). MKLP2 directly binds the N-terminal region of INCENP (Kitagawa et al., 2014). Upon anaphase onset, reversing Cdk1/cyclin B1-dependent phosphorylation of MKLP2 is also essential for CPC relocation to the cell equator and for cytokinesis (Kitagawa et al., 2014), which is similar to reversing Cdk1 phosphorylation of INCENP on Thr59 (Hummer and Mayer, 2009). The reason why Cdk1/cyclin B1 phosphoregulates both MKLP2 and INCENP is unclear. Notably, as Aurora B activity is also required for CPC relocation (Xu et al., 2009), inhibiting Aurora B activity also traps MKLP2 on anaphase chromosomes together with the CPC (Kitagawa et al., 2013, 2014). Thus, it may be possible that Aurora B phosphorylation directly or indirectly activates the motor activity of MKLP2, or it may dissociate the CPC-bound MKLP2 from anaphase chromosomes. In addition, as discussed above, ubiquitination of Aurora B facilitates the active removal of Aurora B (and likely the CPC) from anaphase chromosomes by the AAA+ ATPase and ubiquitin-dependent chaperone p97 (Ramadan et al., 2007). However, it is unclear whether MKLP2 and Cdc48/p97 act in the same pathway for CPC relocation or whether they function independently to remove the CPC from anaphase chromosomes. Interestingly, in KLHL21-depleted cells, MKLP2 localization to the spindle midzone is also reduced (Maerki et al., 2009), which indicates a potential interdependency between MKLP2 and Cdc48/p97 in promoting CPC relocation.

In *S. cerevisiae*, which lack MKLP2, Cdc28 (Cdk1 homolog in budding yeast)-mediated phosphorylation of Ipl1/Aurora B suppresses its association with the microtubule plus-end tracking protein Bim1, which is a homolog of end-binding 1 (EB1). This also inhibits Ipl1/Aurora B localization to the spindle midzone before anaphase onset (Zimniak et al., 2012). In addition, Ipl1/Aurora B relocation also depends on Cdc14 phosphatase, which dephosphorylates multiple Cdc28 and Ipl1/Aurora B sites mainly in the microtubule binding domain of Sli15/INCENP because phosphorylation of these sites inhibits microtubule binding of Sli15/INCENP prior to anaphase onset (Pereira and Schiebel, 2003; Mirchenko and Uhlmann, 2010; Nakajima et al., 2011). It is unclear whether this mechanism is also conserved in mammalian cells (Mocciaro et al., 2010), although the increase in microtubule binding affinity of the CPC is observed by dephosphorylation of Thr59 in INCENP (Hummer and Mayer, 2009).

### The role of CPC relocation as a surveillance mechanism that coordinates mitotic exit, anaphase progression and cytokinesis

Anaphase onset before the completion of metaphase gives rise to segregation errors, whereas the start of cytokinesis before the clearance of trailing chromosomes from the ingressing cleavage plane gives rise to DNA damage (Janssen et al., 2011). The mechanisms how these events are temporally coordinated have just begun to emerge, but CPC relocation is clearly a key regulatory and surveillance mechanism for orderly mitotic exit by serving dual purposes: (1) decreasing Aurora B activity from segregating anaphase chromosomes and (2) increasing Aurora B activity in the cell equator and the midbody.

#### The Role of CPC Relocation in Preventing SAC Reactivation and Mitotic Spindle Instability

The CPC at the inner centromere is required to destabilize the kinetochores improperly attached to mitotic spindles lacking inter-kinetochore tension and to activate SAC that promotes proper chromosome alignment before anaphase onset (Funabiki and Wynne, 2013). In contrast, decreasing inter-kinetochore tension upon loss of centromeric cohesin by separase cleavage may cause undesirable destabilization of mitotic spindles attached to the kinetochores of anaphase chromosomes and reactivation of SAC in anaphase. Therefore, CPC relocation from the centromere upon anaphase onset has been thought to be an effective means of preventing such potentially deleterious events. Indeed, both in *S. cerevisiae* and mammalian cells, failure to remove the CPC from the centromere after anaphase onset (e.g., preventing CPC translocation by depleting MKLP2, blocking PP1-mediated dephosphorylation of histone markers) resulted in the recruitment of mitotic checkpoint proteins, including Mad1, BubR1, and Bub1, to the anaphase kinetochore (Mirchenko and Uhlmann, 2010; Vazquez-Novelle and Petronczki, 2010). However, this re-engagement of SAC components to the kinetochores of anaphase chromosomes does not produce a functional SAC signal that is sufficient to inhibit the anaphase-promoting complex (APC^Cdc20^) because segregation of sister chromatids and APC^Cdc20^-mediated cyclin B degradation occur with normal kinetics without affecting the stability of mitotic spindles attached to the kinetochores (Vazquez-Novelle and Petronczki, 2010). In contrast, the depletion of two PP1-targeting subunits, Sds22 and Repo-Man, which counteract Aurora B phosphorylation of the outer kinetochore component Dsn1 in anaphase, causes transient pauses during poleward chromosome movement, suggesting that removing the CPC indeed contributes to stabilizing the kinetochore–microtubule interface during chromosome segregation (Wurzenberger et al., 2012). However, chromosome segregation in MKLP2-depleted cells is not impaired, indicating that retention of the CPC at the centromeres of segregating chromosomes in anaphase is insufficient to destabilize the kinetochore–microtubule attachments required for chromosome segregation (Vazquez-Novelle and Petronczki, 2010), which may be due to the counteracting PP1 activity at the centromere in anaphase.

#### The Role of CPC Relocation in Controlling Chromosome Condensation Status

**Figure 3:**
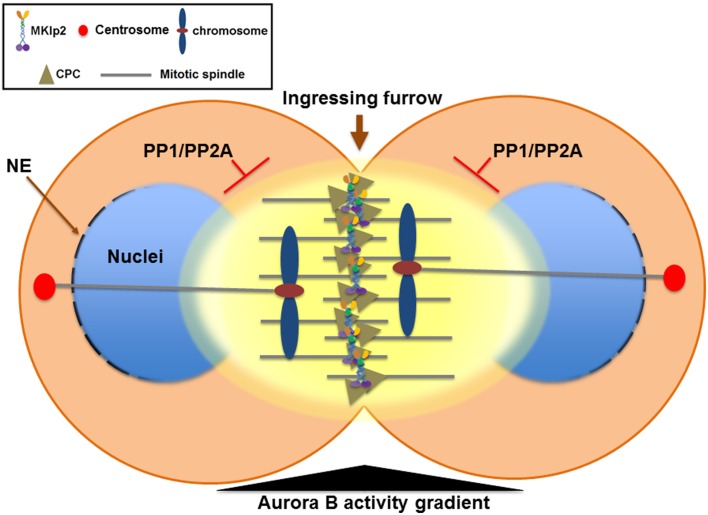
**The Aurora B phosphorylation gradient condenses chromosomes lagging close to the cleavage furrow and delays nuclear envelope reformation (NER) during mitotic exit**. During mitotic exit, partitioning of anaphase chromosomes to the opposite spindle poles requires sister chromatids to be condensed enough to allow their segregation away from the ingressing cleavage furrow. The Aurora B phosphorylation gradient (yellow) is centered at the spindle midzone. Aurora B activity emanating from the spindle midzone promotes hyper-condensation of trailing and lagging chromosome arms until they are cleared away from the ingressing cleavage furrow via phosphorylation of histone H3 on Ser10 and the condensin I complex. Therefore, the CPC relocated to the spindle midzone provides a surveillance mechanism to prevent premature decondensation of trailing and lagging chromosomes. Furthermore, NER is inversely correlated with Aurora B activity on anaphase chromosomes and with the proximity of the spindle midzone. Thus, CPC relocation to the cell equator delays NER near the spindle midzone while it promotes NER near the spindle poles. In contrast, NER occurs simultaneously on all segregating chromosomes if Aurora B is retained on anaphase chromosomes or by global inhibition of Aurora B activity. Furthermore, CPC relocation from anaphase chromosomes to the spindle midzone also serves as a conserved feedback regulator that delays NER in response to incomplete chromosome separation, which may allow for the correction and reintegration of lagging chromosomes into the main nuclei before the completion of NER, thereby preventing micronuclei formation. PP1 and PP2A phosphatases are required for counteracting Aurora B activity to promote NER. NE, nuclear envelope.

The CPC also plays a critical role in promoting chromosome condensation from mitotic entry by recruiting condensin, a conserved protein complex that functions in chromosome condensation and segregation, to nuclear chromatin (Ono et al., 2004; Lipp et al., 2007; Nakazawa et al., 2011; Tada et al., 2011). In contrast, when the sister chromatids start their journey to the opposite spindle poles in anaphase, the CPC is relocated to the cell equator and the segregating sister chromatids undergo chromosome decondensation to re-establish a functional interphase nucleus. Therefore, instead of eliminating Aurora B activity at the centromere of anaphase chromosomes, removing the CPC from the chromosome arm may play an important role in the timely decondensation of anaphase chromosomes and reformation of the nuclear envelope by decreasing Aurora B activity at nuclear chromatin (Figure 3).

#### The CPC at nuclear chromatin in chromosome condensation

During entry into mitosis, chromatin is organized as a highly compacted structure known as mitotic chromosomes. The detailed mechanisms of organizing mitotic chromosomes have recently been reviewed (Thadani et al., 2012). One proposed function of the CPC in mitotic chromosome compaction at the entry into mitosis is regulating the binding of condensin to nuclear chromatin. There are two forms of condensin, condensin I and condensin II, in mammalian cells. They exist as pentameric complexes composed of the structural maintenance of chromosome SMC2 and SMC4 ATPases and three auxiliary subunits (CAP-G/G2, CAP-D2/D3 and kleisin subunit CAP-H/H2) (Ono et al., 2003; Hudson et al., 2009). Overall condensin association with nuclear chromatin is governed by Aurora B localization and activity (Ono et al., 2004; Lipp et al., 2007; Nakazawa et al., 2011; Tada et al., 2011). In *S. pombe*, the CPC member Cut17/Bir1/Survivin, which is essential for the proper localization of the Aurora B-like kinase Ark1, is a nuclear protein in interphase that is required for condensin recruitment to the mitotic nucleus and chromosome condensation (Morishita et al., 2001) while Ark1 activity is required for the mitotic chromatin association of condensin (Petersen and Hagan, 2003; Nakazawa et al., 2008). In *Drosophila* S2 cells, RNAi against Aurora B induces a loss of chromatin-bound kleisin I/Barren, which causes defects in chromosome condensation and segregation, leading to cytokinesis failure and polyploidy formation (Giet and Glover, 2001). In *S. cerevisiae*, condensation of rDNA arrays in anaphase requires Ipl1/Aurora B (Lavoie et al., 2004). Similarly, in *C. elegans*, RNAi against Aurora B inhibits SMC2/MIX1 from being recruited to chromatin (Kaitna et al., 2002). In HeLa cells, RNAi against Aurora B or treatment with the Aurora B inhibitor hesperadin causes a loss of chromatin association of condensin I, but not condensin II (Lipp et al., 2007). Maximal compaction of anaphase chromosomes in rat kidney cells also requires Aurora B (Mora-Bermudez et al., 2007). The association of condensin I to chromatin is also reduced after immunodepletion of Aurora B from *Xenopus* egg extracts (Takemoto et al., 2007). In *S. pombe*, Aurora B-like kinase Ark1 phosphorylates the kleisin protein Cnd2 of condensin throughout mitosis (Nakazawa et al., 2011). Phosphorylation of the human Cdn2 homolog CAP-H by Aurora B promotes efficient association of condensin I, but not condensin II, to mitotic chromosomes in mammalian cells (Ono et al., 2004; Lipp et al., 2007; Tada et al., 2011). Aurora B-dependent phosphorylation of Cnd2 promotes its association with histone H2A and H2A.Z (Tada et al., 2011). The conservation of phosphorylation-dependent condensin interactions with histone H2A variants in *S. pombe* and mammalian cells (Tada et al., 2011) suggests that it is a fundamental mechanism shared in all eukaryotes. Together, it is clear that the CPC plays a critical role in promoting chromosome condensation for mitotic entry.

#### The role of CPC relocation in coordinating proper anaphase chromosome segregation with decondensation

In contrast to the mechanism of chromatin condensation, little is known about what controls chromatin decondensation after the exit from mitosis and in the early G1 phase. In *S. cerevisiae*, Cdc14 phosphatase activity impairs the association of the Cnd2 homolog Brn1 with chromatin (Varela et al., 2009), suggesting that condensin dephosphorylation by Cdc14 promotes chromosome decondensation at mitotic exit. Consistent with this idea, in mammalian cells, PP2A dephosphorylates the CAP-H2 subunit of condensin II during anaphase (Yeong et al., 2003; Takemoto et al., 2009). PP1 also promotes chromosome decondensation because the disruption of mitotic chromosomes in DT40 cells with a conditional knockout of SMC2 of the condensin complex during anaphase can be overcome if Repo-Man is prevented from targeting PP1 to chromosomes (Vagnarelli et al., 2006). Because phosphorylation generally appears to stimulate the biochemical activity of the condensin complex, such as DNA binding and supercoiling, whether its dephosphorylation may reverse these effects to permit chromosome decondensation as cells return to interphase needs to be investigated. In this sense, relocating the CPC from the anaphase chromosome arm to the cell equator is likely important for efficient chromosome decondensation, not only by reversing Aurora B phosphorylation of condensin by PP1 (and also likely PP2A) but also by preventing Aurora B re-phosphorylation of condensin on anaphase chromosomes.

However, this process of decondensation must occur in a tightly regulatory manner because partitioning of anaphase chromosomes to the opposite spindle poles requires sister chromatids to be condensed enough to allow their segregation away from the ingressing cleavage furrow. Moreover, the central spindle must also elongate enough to segregate even the longest chromosomes before chromosome decondensation occurs. Interestingly, the deposition of condensin onto chromosome arms reaches a peak during anaphase when the CPC relocates from anaphase chromosomes to the spindle midzone (Tada et al., 2011). During anaphase, an Aurora B phosphorylation gradient is thought to be centered at the spindle midzone (Fuller et al., 2008) and Aurora B phosphorylation of condensin keeps the segregating chromosomes apart during telophase (Nakazawa et al., 2011; Tada et al., 2011) (Figure 3). An Aurora B phosphorylation gradient emanating from the spindle midzone has been proposed to promote hyper-condensation of trailing and lagging chromosome arms (Neurohr et al., 2011; Tada et al., 2011). In *S. cerevisiae*, Ipl1/Aurora B activity at the spindle midzone phosphorylates histone H3 on Ser10, keeping the trailing anaphase chromosome hyper-condensed until the chromosome has been cleared away from the spindle midzone (Neurohr et al., 2011). Similarly, in *Drosophila* S2 cells while Cdn2 homolog Barren disappears from anaphase chromosomes as sister chromatids separate, Barren is enriched on lagging chromosomes near the spindle midzone and Aurora B activity is required, which is counteracted by PP1 and PP2A activity (Afonso et al., 2014). Therefore, in addition to removing the CPC from anaphase chromosomes to allow for decondensation, the CPC relocation to the spindle midzone appears to actively mediate a surveillance mechanism by retaining condensin to prevent the decondensation of trailing and lagging chromosomes near the ingressing cleavage furrow (Figure 3). Collectively, CPC relocation may ensure that the level of chromosome decondensation is controlled until an effective separation of sister chromatids is achieved.

#### The Role of CPC Removal from Anaphase Chromosomes in Nuclear Envelope Reformation (NER)

Although the mechanism of chromosome decondensation with NER during mitotic exit is not well understood, the CPC also has a critical function in coordinating these two events. Following Cdk1 inactivation and extraction of poly-ubiquitylated Aurora B from anaphase chromosomes by Cdc48/p97 (Ramadan et al., 2007; Meyer et al., 2010), the reassembly of the nuclear pore complex (NPC) starts on the periphery of the segregating chromatin in an ordered step-wise manner (Dultz et al., 2008; Guttinger et al., 2009). In both *Xenopus* egg extracts containing sperm chromatin and *C. elegans* embryos, NER is impaired when Aurora B cannot be extracted from chromatin upon knockdown of Cdc48/p97 (Ramadan et al., 2007), suggesting that, in addition to chromosome decondensation, removing the CPC from anaphase chromosomes might also be important for NER in telophase. In *Drosophila* S2 cells, NER is inversely correlated with Aurora B activity on anaphase chromosomes and with the proximity of the spindle midzone because NER of either laser microsurgery-generated acentric chromosome fragments or lagging chromosomes is significantly delayed compared to the main nuclei formed from efficiently segregated sister chromatids (Afonso et al., 2014). In contrast, NER occurs simultaneously on all segregating chromosomes if Aurora B is retained on anaphase chromosomes by RNAi against MKLP2 homolog Subito or by global inhibition of Aurora B activity (Afonso et al., 2014). Moreover, PP1 and PP2A phosphatases are required for counteracting Aurora B activity to promote NER. Therefore, CPC relocation from anaphase chromosomes to the spindle midzone also serves as a conserved feedback regulator that delays NER in response to incomplete chromosome separation. This feedback mechanism may allow for the correction and reintegration of lagging chromosomes into the main nuclei before the completion of NER, thereby preventing micronuclei formation (Figure 3). However, it is debatable whether this surveillance mechanism functions as the chromosome separation checkpoint as suggested (Afonso et al., 2014) or as part of the sequential mitotic exit events mediated by antagonizing phosphatases (Bouchoux and Uhlmann, 2011). Nonetheless, similar to controlling the timing of chromosome decondensation by the phosphorylation of the condensin I complex, balancing the levels of Aurora B activity on anaphase chromosomes and in the spindle midzone via controlling CPC relocation may be a key determinant of the timing of NER in concert with chromosome condensation status by direct phosphorylation of substrates involved in nuclear envelope disassembly at different stages during mitotic exit.

#### The Role of CPC Relocation in Cleavage Furrow Ingression and Cytokinesis Completion

CPC relocation from anaphase chromosomes to the cell equator is also important for the cytoskeletal reorganization by the CPC that is necessary for cleavage furrow ingression and completion during cytokinesis (Figure 2). Upon anaphase onset, a population of the CPC transfers to the spindle midzone. Shortly thereafter, the CPC also localizes to the equatorial cortex close to the plasma membrane where the cytokinetic machinery is assembled (Earnshaw and Cooke, 1991; Murata-Hori and Wang, 2002). CPC localization to the equatorial cortex also requires MKLP2 (Kitagawa et al., 2013). Communication between the spindle midzone and the equatorial cortex for cleavage furrow ingression may directly proceed along actomyosin filaments as shown in a chemically induced monopolar mitosis (Hu et al., 2008; Kitagawa et al., 2013) and/or occur indirectly by a phosphorylation gradient of Aurora B around the spindle midzone that creates a diffusible signal transmission from the spindle midzone to the equatorial cortex (Fuller et al., 2008). Further studies are needed to clarify the mechanisms of cell division plane specification by which the CPC mediates the communication between the spindle midzone and the equatorial cortex for furrow ingression. Nonetheless, Aurora B activity is required for furrow ingression and completion. The inhibition of Aurora B activity by microinjection of an antibody or hesperadin treatment before the onset of cleavage furrow ingression completely prevents ingression, resulting in binucleation while the inhibition of Aurora B activity after ingression causes regression of the cleavage furrow although the ingression lasts for some time (Guse et al., 2005; Ahonen et al., 2009). Together, continuous Aurora B activity at the cell equator is required for the initiation and robust ingression of the cleavage furrow until completion of stable midbody formation.

The CPC also plays an important role in directly generating and/or maintaining a stable spindle midzone and midbody, which requires the action of the microtubule bundling protein PRC1, the kinesin KIF4 and the centralspindlin complex formed by MKLP1 and Rho GTPase activating protein MgcRacGAP (Kuriyama et al., 2002; Matuliene and Kuriyama, 2002; Mishima et al., 2002; Mollinari et al., 2002; Kurasawa et al., 2004). The CPC is required for the localization of centralspindlin to the spindle midzone (Kaitna et al., 2000). Aurora B phosphorylation of MKLP1 promotes centralspindlin clustering and its microtubule bundling activity (Douglas et al., 2010). Aurora B also phosphorylates and regulates kinesins KIF2a and KIF4 that are implicated in regulating central spindle size (Nunes Bastos et al., 2013; Uehara et al., 2013) while PP2A-B56γ and −ε play a role opposing Aurora B at the spindle midzone, which includes dephosphorylation of the Aurora B phosphorylation site on Thr799 of KIF4A (Bastos et al., 2014). In addition to relocating the CPC to the spindle midzone where Aurora B phosphorylates its substrates, MKLP2 can multimerize with itself and bundle microtubules via its unstructured basic C-terminal stretches (Lee et al., 2010; Kitagawa et al., 2014). Therefore, in addition to its essential role in CPC relocation, MKLP2 may also directly stabilize the spindle midzone and midbody by bundling the anti-parallel microtubules of the central spindle overlap. RNAi against MKLP2 causes binucleation (Hill et al., 2000), which may also be due to a failure in maintaining a stable spindle midzone and midbody. Further details on how the CPC controls contractile ring formation and cleavage furrow ingression for cytokinesis have recently been reviewed (Carmena et al., 2012).

#### The Role of CPC Relocation in Controlling Abscission Timing and Checkpoint

Upon completion of cleavage furrow ingression, the CPC is enriched in the midbody connecting the two daughter cells. The CPC plays a key role in determining the timing of abscission, the final step of cytokinesis, by severing the membrane tether in the midbody. It is now recognized that abscission is a complex process requiring tight spatiotemporal regulation of its machinery to ensure the proper distribution of segregated chromosomes and cytoplasm content between the daughter cells. A group of proteins known as the ESCRT machinery, which mediates the membrane scission process involved in virus budding and a series of common “inward” topology vesiculation events, is also essential for abscission in cytokinesis (Dukes et al., 2008; Elia et al., 2011; Agromayor and Martin-Serrano, 2013). In particular, ESCRT-III subunits form filaments at the plasma membrane of the midbody, which curves inwards, progressively reducing the membrane neck for fission by AAA ATPase vacuolar protein sorting 4 (VPS4), the enzymatic component of the ESCRT machinery (Dukes et al., 2008; Elia et al., 2011; Agromayor and Martin-Serrano, 2013).

**Figure 4:**
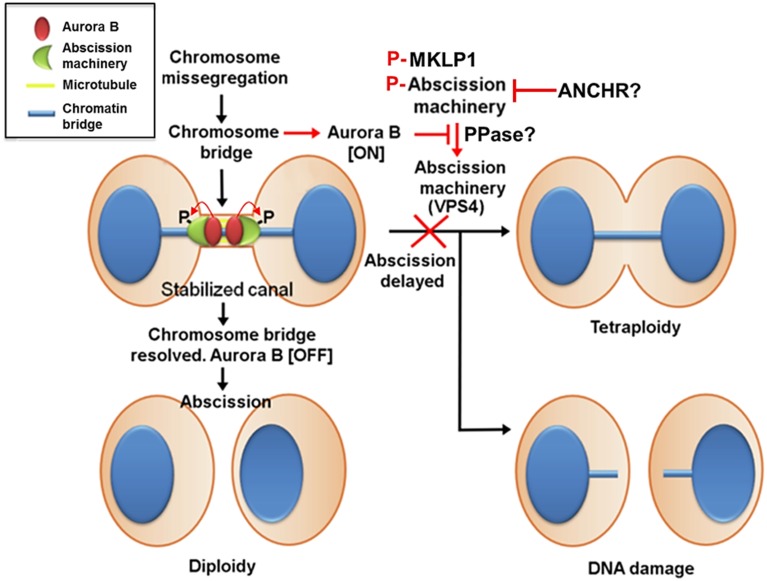
**Aurora B activity suppresses breakage of chromosomes trapped in the midbody and stabilizes the midbody to prevent tetraploidization caused by furrow regression**. In contrast to cleavage furrow formation and ingression, the inhibition of Aurora B activity at the stage of abscission facilitates fission of the intercellular bridge, indicating that Aurora B activity must decrease enough to allow abscission to occur. This mechanism may also prevent chromosome breakage and protect cells from tetraploidization. This delay in abscission (called the abscission checkpoint) requires sustained Aurora B activity, and its downstream targets include MKLP1 and the ESCRT component CHMP4C. Aurora B phosphorylation of MKLP1 seems to stabilize the integrity of the midbody and intercellular bridge. Aurora B phosphorylation of CHMP4C on Ser210 imposes an abscission delay in response to a chromosome bridge that is trapped in the midbody. In concert with CHMP4C, ANCHR prevents VPS4 relocalization from the midbody ring to the abscission zone while it is relieved following the inactivation of Aurora B, thereby promoting membrane scission. However, it is unclear whether MKLP1, CHMP4C, and ANCHR-VPS4 act in the same pathway or whether they function independently downstream of Aurora B activity. Additionally, whether phosphatases antagonize Aurora B activity to promote abscission remains unknown.

Aurora B activity controls abscission timing, which is also proposed to function as a checkpoint that delays abscission in response to a chromosome trapped in the intercellular bridge and is therefore called the abscission checkpoint (similar to the NoCut pathway in yeast) (Norden et al., 2006; Steigemann et al., 2009) (Figure 4). In contrast to cleavage furrow formation and ingression, the inhibition of Aurora B activity at the stage of abscission facilitates fission of the intercellular bridge, indicating that Aurora B activity must decrease enough to allow abscission to occur (Steigemann et al., 2009), which may prevent chromosome breakage and protect cells from tetraploidization. However, the abscission checkpoint is apparently not a failsafe mechanism because missegregating and lagging chromosomes are frequently damaged during cytokinesis, triggering a DNA double-strand break response in the respective daughter cells, which can result in structural chromosome aberrations (Janssen et al., 2011). Nonetheless, this delay in abscission requires sustained Aurora B activity and its downstream phosphorylation targets include MKLP1 and the ESCRT component CHMP4C (Steigemann et al., 2009; Capalbo et al., 2012; Carlton et al., 2012). Aurora B phosphorylation of MKLP1 on Ser911 seems to stabilize the integrity of the midbody and intercellular bridge (Steigemann et al., 2009). CHMP4C engages with the CPC via its interaction with Borealin, which in turn leads to Aurora B phosphorylation of CHMP4C on Ser210 (Capalbo et al., 2012; Carlton et al., 2012). Overexpression of a phosphoresistant mutant after depletion of endogenous CHMP4C fails to impose an abscission delay in response to a chromosome bridge that is trapped in the midbody. In concert with CHMP4C, ANCHR (Abscission/NoCut Checkpoint Regulator; ZFYVE19) is proposed to be a regulator of the abscission checkpoint by its interaction with VPS4 in an Aurora B-dependent manner (Thoresen et al., 2014). ANCHR prevents VPS4 relocalization from the midbody ring to the abscission zone while it is relieved following the inactivation of Aurora B, thereby promoting membrane scission. However, it is unclear whether MKLP1, CHMP4C, and ANCHR-VPS4 act in the same pathway or whether they function independently downstream of Aurora B activity.

Similar to a chromosome bridge trapped in the midbody that delays abscission, the nuclear basket proteins NUP50 and NUP153 provide a link between NPC reassembly and an Aurora B-mediated abscission delay (Mackay et al., 2009, 2010). RNAi against NUP50 or NUP153 not only leads to the mislocalization of multiple NPC components from the nuclear envelope but also delays abscission. The mislocalized NPC components in the cytoplasm perturbs Aurora B targeting to the midbody during cytokinesis (Mackay et al., 2010). Notably, mislocalized Aurora B foci from the midbody do not contain INCENP (also presumably the other CPC components), but the inhibition of Aurora B activity restores abscission (Mackay et al., 2010), suggesting that this delay in abscission is due to sustained Aurora B activation independent of forming the CPC in response to defects in NPC assembly. An important question that remains to be resolved is how apparently different defects in the reforming NPC and the clearing of chromosome bridges trapped in the midbody can communicate to sustain Aurora B activity enough to delay abscission. Finally, recent findings suggest that ESCRT-III assembly and abscission can be induced by tension release in the intercellular bridge when daughter cells have attached to a substrate (Lafaurie-Janvore et al., 2013). This mechanism may allow daughter cells to remain connected until they have settled in their final locations. It remains to be addressed whether Aurora B also governs the abscission timing that is induced by the release of tension, and if so, how it senses the tension imposed on the intercellular bridge.

The phosphorylation status of Aurora B and its substrates at a certain location in the cell is further dictated by phosphatases that counteract Aurora B. In mammalian cells, Aurora B activity is mainly counteracted by PP1 and PP2A (Barr et al., 2011), and the regulatory subunits of these phosphatases that promote their specific localization and define substrate specificity are beginning to emerge, particularly for chromosome congression (Kim et al., 2010; Liu et al., 2010; Foley et al., 2011; Suijkerbuijk et al., 2012; Kruse et al., 2013; Xu et al., 2013, 2014), SAC silencing (Espert et al., 2014), midzone spindle turnover (Bastos et al., 2014), and NER (Vagnarelli et al., 2011). Clearly, the correct balance between kinase and phosphatase activities is crucial for the proper function of the CPC as elevated Aurora B activity causes a failure to stabilize correctly attached spindle microtubules and mitotic dysfunction (Ricke et al., 2011) while too little kinase activity increases the chance of kinetochore-microtubule attachment errors, weakens SAC and shortens abscission timing. Analogous to the action of phosphatases countering Aurora B activity during early and late mitotic events, it is highly likely that such an antagonistic relationship between Aurora B and phosphatases is also present in regulating abscission timing, which is an important topic for further investigation.

### A potential role of the CPC in tumorigenesis and as an anti-cancer therapeutic target

The medicinal properties of the so called anti-microtubule drugs (e.g., paclitaxel) that directly bind and inhibit tubulin have been appreciated for quite some time. Dividing tumor cells need dynamic microtubules to divide, explaining why the anti-microtubule drugs are effective against cancer cells. However, because microtubules are also required for numerous other cellular functions, the anti-microtubule drugs are often toxic to both dividing and non-dividing normal cells. Another common cytotoxin, including DNA damaging drugs, a typical toxicity toward normal dividing cells, is myelosuppression, which is generally reversible and therefore clinically manageable. However, atypical side effects associated with anti-microtubule drugs include peripheral neuropathies, caused by the inhibition of microtubule-dependent processes in neuronal axons and glial cells. Notably, clinically relevant concentrations of paclitaxel cause death in interphase only after a perturbed mitosis (Zasadil et al., 2014), indicating that mitotic aberration is a prerequisite for anti-tumor activity of anti-microtubule drugs. Therefore, it has been anticipated that anti-mitotic agents that prevent mitotic progression without affecting microtubules in non-dividing cells should retain anti-tumor activity without the associated neuropathies.

In this sense, Aurora kinases have been suggested to be promising targets for cancer therapy based on their frequent overexpression in a variety of tumors (Andrews, 2005). Aurora B is also overexpressed in many human tumors, which is thought to result in multinucleation and polyploidy (Nguyen et al., 2009; Dennis et al., 2012). Supporting the contribution of Aurora B to tumorigenesis, its overexpression induced tetraploidy of murine epithelial cells and tumorigenesis in recipient mice (Nguyen et al., 2005, 2009) and increased metastasis of implanted tumors in nude mice (Ota et al., 2002). How Aurora B kinase overexpression facilitates tumorigenesis is an interesting question, and it likely involves genomic instability and tetraploidization, which may fuel to tumorigenesis (Fujiwara et al., 2005).

In proliferative cancer cells, treatment with Aurora B inhibitors induces failed cytokinesis that produces enlarged polyploid cells with multiple centrosomes. After removing drugs, it is expected that these cells proceed to mitosis in a highly uncoordinated manner, leading to unrepairable chromosomal damages and subsequent cancer cell death. Indeed, Aurora B inhibitors are highly effective at killing cancer cells *in vitro* and xenografts in rodent model systems. Notably, in clinical trials, Aurora B inhibitor AZD1152 (barasertib), as a single agent in acute myeloid leukemia (AML) (Löwenberg et al., 2011), showed reasonable responses in approximately 25% of the patients without a significant neuropathy. AZD1152 is currently being evaluated in a Phase III trial in combination with other chemotherapeutic drugs (Marzo and Naval, 2013), and it warrants further evaluation in other hematological malignancies. However, solid tumors fail to show a significant response to AZD1152 (Boss et al., 2011). In contrast to preclinical studies, the lack of solid tumor responses may reflect discrepancies in the growth rate: tumors have an extremely high proliferation rate in preclinical models, which perhaps makes them more susceptible to the actions of AZD1152, whereas the growth rate is slower in solid tumors in patients. Therefore, clinical challenges remain to determine which tumor type(s) from which tissues of origin will be most likely to respond to Aurora B inhibitors and what other genetic or environmental factors contribute to the biological responses (e.g., cell cycle arrest in pseudo-G1, mitotic catastrophe, apoptosis, endoreduplication, cellular senescence, etc.) of tumor cells. Furthermore, in contrast to preclinical studies, it is unclear whether a biologically effective dose has been achieved in the given tumors with optimal treatment schedules and whether the use of validated biomarkers (e.g., phosphorylation of histone H3 on Ser10) in solid tumors can be achieved.

## Future prospective

Mitotic exit is a complex transition involving many dramatic cellular changes to occur in a coordinated manner. For instance, the premature decondensation of sister chromatids, before they are sufficiently removed from the ingressing cleavage furrow, causes chromosome missegregation and breakage. Such defects lead to micronuclei formation, chromosome rearrangement and DNA damage that are often found in human solid tumors. In addition to what it was known about the formation and ingression of the cleavage furrow during cytokinesis after relocating to the cell equator, the emerging view is that the CPC extensively functions in safeguarding genome integrity by monitoring the mitotic exit events so they are executed in an orderly manner. The recently proposed Aurora B-mediated checkpoints during mitotic exit and cytokinesis require further validation, but it is clear that targeting the CPC to different locations at different times during mitotic exit provides dividing daughter cells with a versatile surveillance system to re-establish a functional interphase nucleus. Future research is needed to investigate whether the CPC actively senses and signals to repair certain abnormalities of segregating sister chromatids or only passively delays an improperly executed mitotic exit event. It also remains to be determined how the CPC integrates and translates multiple phosphorylation events to determine the timing of abscission. Furthermore, the phosphatases and the regulatory subunits that counteract Aurora B activity at specific locations with defined substrate specificity are beginning to emerge, particularly before mitotic exit. Clearly, the correct balance between Aurora B and its counteracting phosphatase activities in time and space must also be crucial for regulating proper mitotic exit and completion of cytokinesis, which is an important topic for future research.

### Conflict of interest statement

The authors declare that the research was conducted in the absence of any commercial or financial relationships that could be construed as a potential conflict of interest.
